# An environmental perspective of energy consumption, overpopulation, and human capital barriers in South Asia

**DOI:** 10.1038/s41598-024-53950-z

**Published:** 2024-02-23

**Authors:** Mohammad Mafizur Rahman, Muhammad Iftikhar ul Husnain, Mohammad Naim Azimi

**Affiliations:** School of Business, University of Southern Queensland, Toowoomba, Australia

**Keywords:** Environmental degradation, Energy consumption, Population, Human capital, South Asia, Climate sciences, Ecology, Environmental sciences, Environmental social sciences

## Abstract

Prior literature is substantive in highlighting the nexus between pollutant and socio-economic predictors; however, the role of human interaction has not been sufficiently explored. Thus, the present study examines the validity of the environmental Kuznets Curve (EKC) hypothesis in the presence of energy consumption, overpopulation, and human capital index in five South Asian countries. It employs fixed effects, random effects, and dynamic panel causality techniques with a set of panel data from 1972 to 2021. The baseline results validate the existence of the EKC hypothesis in the recipient panel. Nevertheless, the findings reveal that energy consumption and population density have positive effects, while human capital has negative impacts on *CO*_2_ emissions. Furthermore, the study observes that energy consumption and per capita GDP have a significant causal link with *CO*_2_ emissions, whereas *CO*_2_ emissions are evident to have causality with population density and human capital index. The results are robust and suggest that the consolidation of an effective regulatory framework and technological improvements are substantial measures to improve environmental quality in South Asia. Moreover, allocating sufficient resources to uplift contemporary educational and health status would be imperative to improving environmental quality as aspired to by the Paris Agreement.

## Introduction

Environmental degradation is one of the most threatening global challenges currently faced by humankind^1^. In recent years, environmentalists and policymakers have paid considerable attention to the growing impacts of greenhouse gases (GHGs) on global warming. The prime focus of all countries, especially developing countries, is on how to increase the wealth of their nation by following the policy of fast economic growth. An increase in a nation’s wealth is highly dependent on the consumption of its natural and intellectual resources. Among others, energy consumption is key to economic expansion as it increases the overall economic output of an economy^2^. Meanwhile, energy consumption leads to increased levels of emissions, which damage environmental quality^3^.

South Asia is one of the susceptible regions to environmental hazards that need critical attention. In the past few decades, there has been remarkable economic growth in South Asian countries, mainly due to industrialization and financial sector development. With an average annual growth rate of 4.92% from 1960 to 2020, the region’s GDP swelled from 190.7 to 3241.9 billion US dollars. From 1961 to 1979, there have been several periods when the world’s growth rate outpaced the growth rate of the South Asian region; however, since 1980 onwards, the average growth rate of this region has been above the world’s growth rate^4^. Despite its impressive growth rate at the global level, South Asia is considered an underprivileged region, home to 40% of the world’s poor^5^, vulnerable to climate change, and the epicenter of ambient air pollution^6^. Furthermore, South Asian nations are signatories to the Paris Agreement and are committed to reducing environmental pollution to the pre-industrial level, i.e., below 2 °C. According to Asadullah et al.^7^, nevertheless, these countries have also set their objectives in line with the Sustainable Development Goals (SDGs) agenda of the United Nations. They simultaneously need to focus on uplifting the contemporary living standards of their populations and reducing poverty and income inequality through effective growth policy formulations. If environmental-friendly growth regimes are ignored, such policies may, perhaps, lead to an increase in concurrent energy consumption that causes higher environmental degradation^8^.

From 1971 to 2014, South Asia’s share of the world’s energy consumption doubled, from 40 to 88%. It is a well-established fact that *CO*_2_ emissions increase because of the increase in non-renewable energy (oil, natural gas, and coal) consumption. Reducing energy consumption is recommended to control environmental damage; however, this is not an easy task as it hurts the economic growth of a country^9^. Furthermore, South Asia is one of the most populous regions on earth, with a high population density. Human resources enter as inputs in the production function and are hence essential for economic growth and development. Goods and services markets expand based on population-led demand. However, a larger population means excessive human activities, which derive higher energy demand and deteriorate the environment by increasing the level of *CO*_2_ emissions^10^.

Prior literature has extensively explored the nexus between environmental quality and economic output through the celebrated Environmental Kuznets Curve (EKC) hypothesis. The EKC assumes that there exists an inverted U-shaped curve between economic output and the indicators of environmental quality^11^. Following the seminal work by Apergis and Payne^11^, voluminous empirical literature^3,12–18^ exists that analyzes the relationship between economic expansion and environmental quality using the EKC’s framework in the context of individual countries or regional panels. Nonetheless, a handful of studies can be listed that have examined the validity of the EKC hypothesis in the context of South Asia^14,19–23^ and have offered conflicting results. We selected South Asia as the theme of this investigation for two reasons: First, the scarcity of studies in the South Asian context has caused policymakers to have little understanding of the sensitivity of compromising environmental quality for the sake of gaining swift economic growth and, thus, have paid little attention to adjusting the existing or formulate new growth-environmental policies. Therefore, the present study is an imperative piece of investigation that adds to the contemporary body of knowledge in relation to the nexus between growth and environmental quality in the presence of human interaction. Second, South Asia is an important area of research; its rapid population growth and swift economic transformation contribute to various environmental problems in overall Asia. They include habitat destruction, pressure on land, significant loss of biodiversity, extreme air pollution, climate change, and global warming.

Moreover, in an empirical sense, the conflicting results of the above-cited studies can be attributed to various factors. Firstly, these divergent results are due to the use of single-country annual data, which is normally limited to 30–40 years. According to DeJong et al.^24^ and Narayan and Smyth^25^, the conventional unit root and cointegration tests lead to spurious results due to their low power in small samples. Secondly, the model used to examine the relationship between environmental quality and economic development is restrictive in nature and may not identify all possible forms of nonlinearity that may exist^26^. Thirdly, panel data regression models are plagued by serious challenges, including time instability and cross-section heterogeneity. Conventional unit root tests produce misleading results in the presence of potential cross-sectional heterogeneity^27,28^. Although means of fixed or random individual effects in combination with time effects are used in empirical literature to overcome this problem, the inadequacy of these methods cannot be ruled out^29^. One way to overcome these potential problems is to use innovative and recently developed econometric methods that cater to time instability and heterogeneity problems commonly found in the baseline model. For instance, the heterogeneous panel causality test of Dumitrescu and Hurlin^30^ is based on the premise that coefficients across cross-sections can vary. Thus, these empirical shortcomings have further motivated us to carry out this work and fill the gaps by providing consistent and robust results that can assist relevant policymakers in South Asia. Although filling the gaps forms part of our motivation, we stir the present study by formulating three specific research questions that explain the overarching objectives of the study as follows: First, is the EKC hypothesis valid in the South Asian context? Second, what is the scale and magnitude of the effects of overpopulation on existing *CO*_2_ emissions? Third, is there any long-run memory between energy consumption, overpopulation, *CO*_2_ emissions, and growth indicators in South Asia? Providing consistent answers to these questions will highlight two specific areas where policy tensions exist: first, the human interaction of the environmental-growth nexus; and second, the environmental growth regime to correspond with the achievement of the committed SDG milestones in South Asia.

The present price of work is a novel investigation in South Asia and contributes to the empirical literature on many fronts. First, according to Dogan and Seker^31^, a major criticism of the existing energy-growth-environment literature is the choice of panel econometric methods that fail to address heterogeneity and cross-sectional dependence that cause forecasting errors. We use Driscoll and Kraay’s^32^ method, which accounts for cross-sectional dependence and hence rules out the possibility of obtaining misleading results. In the same vein, this study employs the dynamic panel causality test, which is superior to other tests of causality. Second, this study is comprehensive in nature as it uses the most recent and extended data available for five South Asian countries (Bangladesh, India, Nepal, Pakistan, and Sri Lanka). Third, we introduce the human capital index, a measure of human interaction, as a predictor of *CO*_2_ emissions. Not many studies have examined the impact of human interaction, even though it can potentially affect the environment. Furthermore, a paradigm shift in climate change policy can be observed since it focuses on overall improvements in social welfare represented by measures of human development instead of a mere increase in GDP growth^33^. According to Costa et al.^34^, human interaction is positively associated with *CO*_2_ emissions. Fourth, even though the nexus between energy consumption and economic growth has been the focus of many empirical studies in the recent past, they have mainly targeted developed countries, including the Organization for Economic Cooperation and Development (OECD) and European countries^35^, while there is little attention given to the South Asian region. This region is on an unsustainable development path as they spend a large proportion of revenues on oil imports instead of investing in human capital.

The remaining parts of this study are organized as follows: “Literature review” Section reviews relevant literature. “Data and Methods” Section explains the data and methodology used to analyze the data. “Empirical findings” Section presents the empirical findings. “Discussion” Section presents a discussion on the findings. “Conclusion and policy implications” Section concludes the study and highlights specific policy implications.

## Literature review

Prior literature documented the influence of various determinants of *CO*_2_ emissions in both developing and developed economies on the environment. Different researchers have used various measures to demonstrate the association between GDP, *CO*_2_ emissions, and environmental degradation. Grossman and Krueger^36^ revealed a rise in pollution with an increase in the per capita GDP of the less developed economies while a fall in pollution with an increase in the per capita GDP of the developed economies. In addition, Panayotou^37^ asserted corrosion in environmental quality with a rise in GDP per capita up to a certain extent with the EKC illustration. With the present consensus regarding the EKC, it is pertinent to investigate the determinants that may slow down or hasten the advent of the inflexion level of the EKC. Environmental sustainability is primarily subject to energy consumption^38^, and expansion in energy consumption does not result in economic growth but mostly becomes a barrier to environmental sustainability^39^. Mukhtarov et al.^40^ reported energy consumption as a worry for environmental quality. Ding et al.^41^ suggested that energy consumption and international trade are the primary contributors of *CO*_2_ emissions in the G7 countries. Ali et al.^42^ used the top 10 carbon-emitter countries and investigated the role of renewable energy, trade, and environmental advancement. The findings from the Westerlund cointegration method revealed a long-term association between renewable energy consumption, trade, and environmental advancement. Sarkodie and Strezov^43^ examined the nexus between CO_2_ emissions and energy consumption in the case of five emerging countries over a period of 34 years. The findings revealed a likely increase in *CO*_2_ emissions in response to an increase in energy consumption.

Nathaniel and Adeleye^44^ conducted a research study with a sample of 44 African countries using ecological footprint and *CO*_2_ emissions as a proxy of environmental degradation from 1992 to 2016. The findings with dynamic and static econometric models revealed that energy consumption with an asymmetric influence of urbanization degrades environmental sustainability. Wang et al.^45^ investigated the role of industrialization, population, and urbanization on environmental dilapidation from 1995 to 2014 in China and revealed industrialization, population, and urbanization as the chief drivers of *CO*_2_ emissions for China. Shahzad^46^ reviewed prior literature for environmental quality, energy consumption, and environmental taxes up to 2020 for both developing and developed economies, with the prime objective of covering different levels of methodologies, modeling, timeframes, and economies in the survey. The study revealed that empirical studies on the subject mostly found a positive influence of energy usage on pollutant emissions, with an ambiguous role for environmental taxes. Table 1 summarizes the most recent studies on the EKC in South Asia.

**Table 1:** Most recent studies on the EKC in South Asia.

Authors	Context	Period	Methods	Key findings
Murshed et al.^57^	South Asia	1972–2014	Panel data analysis	EG and EX affect *CO*_2_
Majumdar et al.^58^	South Asia	1972–2015	Quantile regression	EN affects *CO*_2_
Khan et al.^59^	South Asia	1972–2017	FMOLS	Bidirectional causality between EGU and EG. Causality between *CO*_2_ emissions and GDP
Lau et al.^60^	ASEAN	2000–2020	Systematic review	Mixed
Sadiq et al.^23^	South Asia	1972–2019	FMOLS	EKC validated
Amin et al.^61^	South Asia	1998–2019	Panel data analysis	FI, GDP, and FDI affect *CO*_2_
Tan et al.^22^	Southeast and South Asia	2013–2019	Binomial regression	EKC validated
Mehmood et al.^20^	South Asia	1995–2014	ARDL	Mixed
Reza et al.^62^	South Asia	1995–2018	FMOLS and DOLS	Mixed
Sharma et al.^63^	South Asia	1990–2016	CS-ARDL	SMT and income affect CO_2_
Murshed and Dao^19^	South Asia	1995–2015	Panel data analysis	IT, RET, EG, and FDI affect EF
Jingpeng et al.^64^	South Asia	1980–2019	Quantile and non-parametric causality	Mixed
Fong et al.^65^	Southeast Asia	1993–2012	Spatial regression	EKC validated
Sattar et al.^66^	South Asia	2004–2019	FMOLS	CODI affect CO_2_
Hanif et al.^67^	ASEAN	1995–2020	Cointegration analysis	NRE and GL affect CO_2_
Rahman and Alam^68^	Asia–Pacific	1960–2020	Driscoll and Kraay’s standard error and PCSE	Mixed
Wangzhou et al.^69^	5 High emitters in Asia	1995–2019	Panel-NARDL-AMG	TO affects CO_2_
Sampene et al.^70^	South Asia	1990–2017	CS-ARDL and Dumitrescu and Hurlin causality	Mixed
Rahman et al.^14^	South Asia	1990–2017	panel cointegration	Mixed

EG: economic growth, EX: export, EL: electricity, FI: financial inclusion, TR: trade openness, EI: economic inclusion, EF: ecological footprints, IT: intra-regional trade, RET: renewable energy transition, CODI: China’s outwards direct investment, NRE: non-renewable energy, TO: tourism.

Growth in energy consumption levels has been coupled to population growth, which, as a result, accelerates the energy sector to increase its capacity. Estimates revealed that the increasing reliance on fossil fuels increases energy consumption. The increase in *CO*_2_ emissions with the increasing demand for fossil fuels is an obstacle to environmental sustainability. It is scientifically proven that the earth’s average temperature is growing in response to increasing GHGs and *CO*_2_ emissions, which eventually lead to environmental degradation^47^. The formation of renewable and environmentally friendly sources for energy is useful for environmental sustainability^48^.

A class of studies also found overpopulation as a barrier to environmental sustainability, with mixed findings in both developing and developed countries^49,50^. Begum et al.^51^ investigated the dynamic influence of overpopulation, energy consumption, and GDP growth on *CO*_2_ emissions using the ARDL bond testing methodology for Malaysia from 1970 to 2009. The findings suggested that both per capita GDP and energy consumption have a long-term positive influence on *CO*_2_ emissions. Moreover, the findings found no significant influence of overpopulation on *CO*_2_ emissions in the case of Malaysia. Rehman et al.^52^ examined the global influence of population growth, globalization, renewable energy, nuclear energy, and economic growth on *CO*_2_ emissions and found that overpopulation and globalization positively influence *CO*_2_ emissions while economic growth negatively impacts *CO*_2_ emissions both in the short and long run. A handful of studies have recognized that most of the environmental barriers are human-induced. In other words, human actions, either unintentional or intentional, mainly caused environmental degradation.

Jahanger et al.^53^ examined the validity of the EKC hypothesis in a panel of top nuclear energy-generating countries. They employed a set of data over the period from 1990 to 2018 and used the dynamic common correlated effects model for analysis. Regardless, the authors found that military expenditures, nuclear energy, and particularly human capital are significant in reducing environmental decay in the recipient panel. Ganda^54^ explored the environmental response to human capital using the more recent CS-ARDL and Dumitrescu-Hurlin’s causality tests for BRICS countries from 1990 to 2017. The study found that human capital significantly influences environmental sustainability and quality in both the long and short run. Çamkaya et al.^55^ examined the long-term consequences of human capital for ecological footprint and *CO*_2_ emissions for Turkey, with additional long-term consequences of financial development, globalization, and GDP for *CO*_2_ emissions. The results obtained from the Fourier FADL approach the unveiled negative consequences of human capital for both EF and *CO*_2_ emissions. The results further indicated a positive influence of financial development and GDP on *CO*_2_ emissions. Abdouli and Omri^56^ explored the nexus between economic growth, human capital, environmental quality, and FDI inflows in the Mediterranean region. The study presented mixed findings, such as bidirectional linkages among FDI, economic growth, human capital, and *CO*_2_ emissions for all the cases except Asian and Euro-Mediterranean economies, unidirectional linkages to FDI inflows from human capital, from economic growth to FDI inflows, and from human capital to *CO*_2_ emissions except for African Mediterranean economies.

## Data and methods

### Data

Based on the availability of the required data, this study employs a set of panel data over the period from 1972 to 2021 relevant to the South Asian countries, namely, Bangladesh, India, Nepal, Pakistan, and Sri Lanka. However, South Asia consists of eight counties, but due to data limitations, we dropped Afghanistan, Bhutan, and the Maldives from the analysis to avoid any potential inconsistency and bias in the subsequent regression analysis. In a bid to examine the validity of the EKC hypothesis in South Asia, the study selects and employs a set of variables that are consistent with prior empirical literature and the primary objectives of this study. *CO*_2_ emissions (*CO*_2_) expressed in metric tons per capita have been used as the dependent variable. Recent studies by Mikayilov et al.^71^, Jahanger et al.^72^, Jayanthi et al.^73^, and Jahanger et al.^74^ have also employed *CO*_2_ as the measure of environmental degradation across different economies. Furthermore, following Aslam et al.^75^, Adeleye et al.^76^, Jahanger et al.^77^, and Ivanovski et al.^78^, per capita GDP (PCGDP), expressed in constant 2015 US$, has been used to measure the economic growth variations passing through different stages of development in South Asia. Energy consumption (ENGU), expressed in kilograms of oil equivalent per capita, has been employed to measure the level of energy being used in response to economic output. Increased energy use, whether for industrial or residential purposes, is expected to result in increased carbon emissions^79,80^. In an effort to address our primary study concerns, we added population density expressed in people per square kilometer of land area to our analysis. Finally, the human capital index has been employed to measure the growth of human interaction with environmental quality^81^. All datasets relevant to the cited variables have been collected from the World Development Indicators (WDI), sources that are relevant to the World Bank Group^82^.

### Methods

The primary objective of this study is to examine how socio-economic and demographic factors (overpopulation, human capital, per capita GDP, and energy consumption) are related to *CO*_2_ emissions in South Asia. We built our work on the EKC hypothesis framework following similar methods used in Rahman and Vu^83^ and Zoundi^84^. The theory posits an inverted U-shaped relationship between pollution and per capita income. Consequently, the present study employs the following functional form of the economic model:

1 $$CO_{2it} = f\left( {ENGU_{it} ,\;PCGDP_{it} ,\;PCGDP_{it}^{2} ,\;POPD_{it} ,\;HCI_{it} } \right)$$

where *CO*_2_ represents carbon emissions per capita, ENGU represents energy consumption, PCGDP refers to per capita gross domestic product, PCGDP^2^ is the square of the per capita GDP, POPD refers to the population density, and HCI indicates human capital index in country *i* and time *t*. The inclusion of PCGDP and PCGDP^2^ in Eq. (1) shows that the EKC is a non-linear inverted U-shaped curve, which posits that pollution first increases with economic development and subsequently falls gradually^85^.

#### Diagnostic tests

To estimate Eq. (1) and avoid any misspecification, there are several preliminary tests that need to be performed. Thus, we start our investigation with the assessment of cross-sectional dependence (CD) in the recipient panel prior to testing for stationarity and long-run cointegration. Overlooking CD in analysis may result in biased outcomes. In the current changing world, economies are interrelated through cultural, financial, and economic ties. Therefore, the accuracy of the estimators may be affected by the spread of shocks from one unit (country) to another. Therefore, to assess CD, we employ the proposed Lagrange multiplier (LM) test of Breusch and Pegan^86^. In contrast to other spatial tests, the LM test does not necessitate for cross-sectional order and, hence, is more commonly applicable with sufficiently large T relative to finite N. Breusch and Pagan’s LM statistics with the null of CD are based on the given equation:

2 $$CD_{BP} = \sum\limits_{i = 1}^{N - 1} {\sum\limits_{j = 1 + 1}^{N} {\hat{p}_{ij}^{2} } }$$

where the residuals’ pair-wise correlation is represented by $\hat{p}_{ij}^{2}$. For cases where T is finite with a sufficiently large N, a new estimator for CD is needed with no dependence on a specified spatial weight matrix^87^. Unlike, the asymptotically distributed LM test as Chi-square, Pesaran^87^ proposed an alternative CD estimator with the null of no CD on the basis of pair-wise correlation suitable for large N and small T as follows:

3 $$CD_{P} = \sqrt {\frac{2T}{{N\left( {N - 1} \right)}}} \left( {\sum\limits_{i = 1}^{N - 1} {\sum\limits_{j = i + 1}^{N} {\hat{p}_{ij}^{2} } } } \right)$$

where the residuals of pair-wise correlation are represented by ${\hat{\text{p}}}_{{{\text{ij}}}}^{2}$. $CD_{p}$ is an estimate of Pesaran’s^87^ method, having an absolutely zero mean for fixed values of T, and N shows its applicability for a wide range of panels, such as panels with heterogeneity (panels with slopes and variances of complex breaks) or static/dynamic panels.

#### Panel unit root tests

Before conducting any panel cointegration analysis, it is necessary to assess the stationarity of a panel series of variables and determine their order of integration. Stationarity indicates that the statistical properties of a panel, such as the mean, variance, and autocorrelation structure, do not change over time. Without a stationary process, the regression would be spurious, necessitating a long-run cointegration test. The study in hand used the first-generation test of Maddala and Wu^88^ for obtaining the stationarity of a variable. Using the p-values obtained from the ADF tests, Maddala and Wu^88^ constructed the following statistical model, with the null hypothesis defining the series as I(1):

4 $$P = - 2\sum\limits_{i = 1}^{N} {\ln \left( {P_{i} } \right) \sim \chi_{2N}^{2} }$$

We also used the cross-sectionally augmented (CIPS) model of Pesaran^89^ for obtaining CD in the variables with the avoidance of size distortion and troubles while detecting the cross-sectional dependence. The second-generation test of CIPS is robust to both heterogeneity and CD, having an alternative of not less than one stationary cross-section versus the non-stationarity of all the cross-sections as the null hypothesis^18^. The obtained CIPS statistic after averaging the CADF is given below:

5 $$CIPS = \left( \frac{1}{n} \right)\sum\limits_{i = 1}^{N} {t_{i} }$$

where $t_{i}$ represents the t-statistics of the $\beta_{i}$’s in the CADF regression.

#### Panel cointegration and causality

Depending on the findings of panel unit root tests (which identify whether the variables of interest are non-stationary), a panel cointegration test must be conducted to assess whether there is a long-run equilibrium relationship between the variables. Therefore, we conducted Kao’s^90^, Pedroni’s^91,92^, and Westerlund’s^93^ panel cointegration tests. Kao’s and Pedroni’s tests assume that the cointegrating vector is the same for all panels; however, Westerlund’s technique permits panel-specific cointegrating vectors. Moreover, Westerlund’s cointegration test utilizes an error correction model (ECM) method to determine if ECM contains error correction. In addition to the panel cointegration test for examining long-run relationships, we undertake the heterogeneous panel causality test of Dumitrescu and Hurlin’s^30^ to explore the short-term bivariate causal link between the variables. This test is based on the premise that coefficients across cross-sections will vary. In order to identify causality in panel data, the following model is used:

6 $$Y_{it} = \alpha_{i} + \sum\limits_{j = 1}^{K} {\delta_{i}^{j} Y_{it - j} + \sum\limits_{j = 1}^{K} {\beta_{i}^{j} X_{it - j} } + \varepsilon_{it} }$$

where $\delta_{i}^{j}$ is the autoregressive parameter and $\beta_{i}^{j}$ is the regression coefficients. The null hypothesis states that there is no causal relationship for any cross-section in the panel.

#### Fixed effects and random effects

In an empirical sense, panel data regression yields consistent estimates. It allows for the mitigation of unobserved individual heterogeneity and omitted variable bias^94^. A general form of the linear panel model is given below:

7 $$Y_{it} = \alpha_{i} + X_{it}^{\prime } \beta + \varepsilon_{it}$$

where $Y_{it}$ is the dependent variable (say, *CO*_2_ emissions), $X_{it}$ refers to a vector of covariates (ENGU, PCGDP, PCGDP^2^, POPD, and HCI), and $\beta$ is the vector of respective regression coefficients. For estimation purposes, we conduct fixed effects and random effects panel regression models. For fixed effects, we define $\tilde{Y}_{it} = Y_{it} - \overline{Y}_{i}$ and $\tilde{X}_{it} = X_{it} - \overline{X}_{i}$ , and run the following panel regression:

8 $$\hat{Y}_{it} = \alpha_{i} + \hat{X}_{it}^{\prime } \beta + \varepsilon_{it}$$

Likewise, for random effects model, we estimate the following regression.

9 $$Y_{it} = \overline{\alpha } + X_{it}^{\prime } \beta + v_{it}$$

with the following additional assumption that $\alpha_{i} \sim \left[ {\alpha , \sigma_{\alpha }^{2} } \right]$ and ${\varvec{\varepsilon}}_{{{\varvec{it}}}} \sim \left[ {o, \sigma_{\varepsilon }^{2} } \right]$. For robustness, we used Driscoll and Kraay’s^32^ standard errors in our panel regressions to address all the issues described in our diagnostic tests. Once both models are estimated, we perform the Hausman test to select the more suitable model^95^.

## Empirical findings

**Figure 1:**
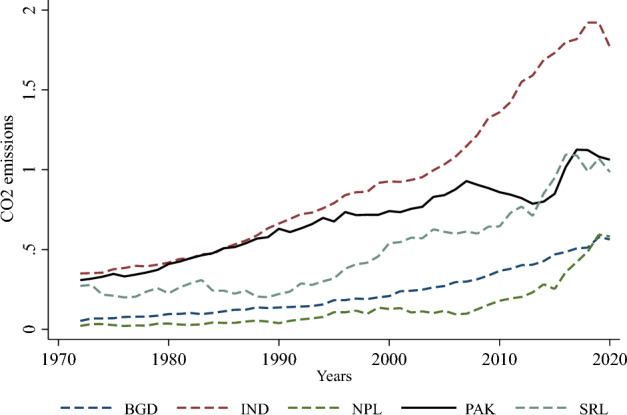
*CO*_2_ emissions by panel countries. Note: BGD: Bangladesh, IND: India, NPL: Nepal, PAK: Pakistan, SRL: Sri Lanka. Source: Authors’ depiction.

**Figure 2:**
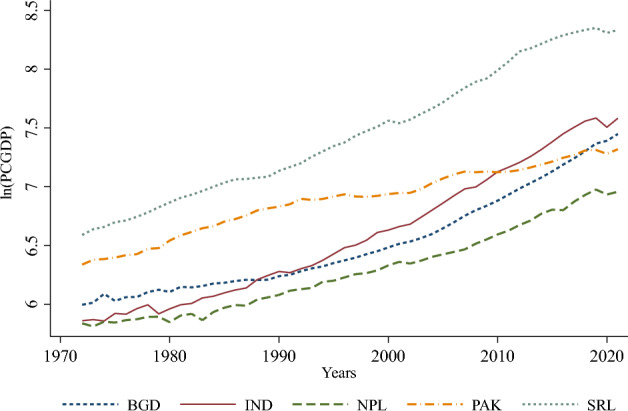
Natural log of PCGDP by panel countries. Note: BGD: Bangladesh, IND: India, NPL: Nepal, PAK: Pakistan, SRL: Sri Lanka. Source: Authors’ depiction.

First, we investigate and characterize the data to identify the underlying pattern of the panel variables used in the study. Table 2 provides a descriptive analysis of the variables and Figs. 1 and 2 depict the trend of CO_2_ emissions and the log of PCGDP. Table 2 reports the mean, standard deviation, minimum, and maximum values for all the variables. It is observed that the annual averaged per capita *CO*_2_ emissions for the countries are 0.495 metric tons with minimum and maximum of 0.021 and 1.922 metric tons, respectively. The notable difference between the maximum and minimum values of *CO*_2_ emissions, with a significant standard deviation of 0.41, can be attributed to time. An increasing trend in *CO*_2_ emissions can be observed in Fig. 1, while an upward trend can also be observed in PCGDP, as shown in Fig. 2. It implies that over the period, both per capita *CO*_2_ emissions and per capita GDP have grown significantly during the period from 1972 to 2021. The reported statistics regarding all the variables are adequate for further empirical analysis without trimming or dropping any of the variables.

**Table 2:** Descriptive statistics.

Variables	Obs	Mean	Std. Dev	Minimum	Maximum
CO_2_ emissions	250	0.495	0.410	0.021	1.922
Energy consumption	250	352.354	144.555	88.147	852.016
Per capita GDP	250	1038.978	783.350	333.900	4228.149
Per capita GDP^2^	250	1,690,658	3,162,253	111,489.3	17,877,244
Population density	250	369.363	300.696	79.626	1277.587
Human capital index	250	1.751	0.514	1.044	2.900

Source: Authors’ calculations.

Second, serial correlation, heteroscedasticity, and cross-sectional dependence for the series are shown in Table 3. Both serial correlation and heteroscedasticity were detected in the series. The table also reports test statistics based on Breusch LM and Pesaran CD tests with p-values. Both the statistics rejected the null of CD among the series, i.e., the obtained p-values based on both the Breusch Pagan LM and Pesaran CD tests rejected the null of no cross-sectional dependence with 99% and 90% confidence, respectively. With the observed CD, panel techniques with heterogeneous properties may be used^18,31^.

**Table 3:** Panel preliminary tests results.

Tests performed	Statistics	*p*-values
Serial correlation	18.445	0.013**
Heteroscedasticity	180.24	0.000***
Breusch-Pagan LM	84.486	0.000***
Pesaran CD	− 1.803	0.071*

*Source*: Authors’ calculations.

*, **, and *** indicate significance at 1%, 5%, and 10% levels, respectively.

Third, in light of the observed serial correlation, heteroscedasticity, and CD among the variables, the use of common panel unit root tests would fail to capture the true stationarity of the variables. Therefore, Table 4 presents the second-generation (CIPS) panel unit root test of Pesaran^89^ in addition to the Maddala-Wu CD test. The CIPS estimator is robust and applicable in cases of both the CD and heterogeneity^18^. To consider long-run decline or growth in the series, a linear time trend (specification with trend) was also used to improve the power of the CIPS test to observe stationary behaviour. Both the Maddala and Pesaran CIPS tests, without and with trend, are insignificant in rejecting the null of non-stationarity. However, Maddala’s test rejects the null of non-stationarity of the POPD at 1% significance; the CIPS reports otherwise. Our conclusion is based on the results of CIPS, and we observe that all the variables are level-stationary; say, they follow the I(0) series.

**Table 4:** Panel unit root tests.

Variables	Maddala-Wu				Pesaran’s CIPS			
	Without trend		With trend		Without trend		With trend	
	Statistics	*p*-values	Statistics	*p*-values	Statistics	*p*-values	Statistics	*p*-values
CO2	0.448	1.000	4.342	0.931	1.623	0.948	2.172	0.985
ENGU	4.706	0.910	0.045	1.000	3.717	1.000	3.076	0.999
PCGDP	0.048	1.000	1.425	0.999	2.726	0.997	2.988	0.999
PCGDP2	0.004	1.000	0.260	1.000	4.012	1.000	5.281	1.000
POPD	8.037	0.625	60.900***	0.000	1.439	0.925	1.374	0.915
HCI	15.815	0.105	8.142	0.615	5.550	1.000	1.084	0.861

*Source*: Authors’ calculations.

***indicates significance at 1% level.

The results obtained in Table 4 suggest to test for the long-run relationship among the variables^96^. Thus, the study employs Westerlund’s cointegration test to delve into the long-run nexus between the variables, while Kao’s and Pedroni’s tests are used for cross-validation. The results of the cointegration analysis are reported in Table 5. The results of Westerlund’s cointegration test, which is appropriate for capturing the true cointegrating vectors of the variables in the presence of cross-sectional dependence, are significant to reject the null of no cointegration at the 5% significant level.

**Table 5:** Cointegration test results.

Tests performed	Westerlund test		Kao test		Pedroni test	
	Statistics	*p*-value	Statistics	*p*-value	Statistics	*p*-value
Variance ratio	− 1.648**	0.049				
Modified Dickey–Fuller t-stat			0.260	0.398		
Dickey–Fuller t-stat			1.076	0.141		
Augmented modified Dickey–Fuller t-stat			− 1.493*	0.068	− 3.612***	0.000
Modified variance ratio					− 1.010	0.156
Modified Phillips–Perron t-stat					− 1.817**	0.035
Phillips–Perron t-stat					− 2.934***	0.002

*Source*: Authors’ calculations.

*, **, and *** indicate significance at 1%, 5%, and 10% levels, respectively. The null hypothesis for all three tests assumes no cointegration.

Moreover, the estimated results of Kao’s test are only significant to reject the null at 10% using the augmented modified Dickey–Fuller statistics; however, Pedroni's test is statistically significant to confirm the results of Westerlund’s and reject the null of no panel cointegration at a significant level of 1%. Thus, the results imply that *CO*_2_ emissions, PCGDP, the square of PCGDP, POPD, ENGU, and HCI have a long-run relationship—that is, they move together in the long run. After the detection of cointegration, it is pertinent to test the causal relationship between the variables^12^ to assist policymakers in framing policies regarding environmental sustainability. The findings based on Dumitrescu-Hurlin’s^30^ causality test are reported in Table 6. We noticed strong evidence of unidirectional causality, such as ENGU, PCGDP, and PCGDP^2^ causing *CO*_2_ emissions, and *CO*_2_ emissions causing POPD and HCI in the case of South Asian countries. Our findings show that PCGDP causes *CO*_2_ emissions that are consistent with prior literature, and the predictive power of energy over *CO*_2_ emissions is consistent with the conclusion reached by^18^. The evidence that *CO*_2_ emissions cause POPD and HCI can be linked to the Boserupian view. The state of environmental sustainability may show negative elasticity or no relationship with population (Boserupian view). Population growth can result in any demographic or 'multiphasic' way, with the possibility of reducing resource pressure.

**Table 6:** Panel causality test.

H0: Xs cause *CO*_2_	W-bar	z-statistics	*p*-value	H0: *CO*_2_ causes Xs	W-bar	z-statistics	*p*-value
ENGU → *CO*_2_	14.763	21.761***	0.000	*CO*_2_ → ENGU	2.235	1.952	0.220
PCGDP → *CO*_2_	7.114	9.667***	0.000	*CO*_2_ → PCGDP	2.247	1.972	0.200
PCGDP^2^ → *CO*_2_	8.313	11.562***	0.000	*CO*_2_ → PCGDP^2^	1.914	1.446	0.220
POPD → *CO*_2_	3.261	3.574	0.420	*CO*_2_ → POPD	58.492	90.902***	0.000
HCI → *CO*_2_	3.375	3.756	0.330	*CO*_2_ → HCI	9.147	12.881***	0.000

*Source*: Authors’ calculations.

*** indicates significance at 1% level. → indicates the direction of causality.

Finally, the study estimates the effects of variables on *CO*_2_ emissions using fixed effects and random effects model with Driscoll and Kraay’s^32^ standard error approach. The results are reported in Table 7. The *p*-value based on Hausman’s test (0.292), which reported underneath Table 7, is not significant to reject the null hypothesis of random effect preference over fixed effects model. It indicates that the residuals are uncorrelated with the cross-sectional effects in the recipient panel, using random effects model. However, the results of Hausman’s test suggest that findings based on the fixed effect would be inconsistent.

**Table 7:** Panel regression results.

Dependent variable: *CO*_2_	Random effects model			Fixed effects model		
	Coefficients	DK-Std. error	t-values	Coefficients	DK-Std. error	t-values
ENGU	0.003***	0.000	8.51	0.002***	0.001	4.53
PCGDP	310.154*	162.390	1.91	429.030**	181.651	2.36
PCGDP^2^	− 0.065**	0.025	− 2.60	− 0.075***	0.026	− 2.87
POPD	0.001***	0.000	5.83	0.000	0.000	0.57
HCI	− 0.227***	0.071	− 3.22	− 0.162*	0.090	− 1.80
Constant	− 0.534***	0.135	− 3.96	− 0.334	0.097	− 3.44
Diagnostic checks						
Adjusted r-squared	0.842			0.911		
F-statistics	437.627***	0.000		46.456***	0.000	
Normality (JQ)	1.6026	0.325		4.057	0.131	

*Source*: Authors’ calculations.

*, **, and *** indicate significance at 1%, 5%, and 10% levels, respectively. DK: Driscoll and Kraay. Hausman’s chi-squared = 2.46 with a *p*-value of 0.292. JQ: Jarque–Bera test.

The estimated coefficient based on both the random effects and fixed effects suggest a significant positive impact of ENGU, PCGDP, and POPD on *CO*_2_ emissions. The significant increase in *CO*_2_ emissions with the increase in PCGDP, ENGU, and POPD confirm the existence of an inverted U-shaped curve, say, the EKC. The inverted U-shaped curve between the subject variables and *CO*_2_ emissions is well documented by Tan et al.^22^. The positive impacts of ENGU, PCGDP, and POPD on *CO*_2_ emissions are consistent with those of Rahman and Vu^85^, Amin et al.^61^, Hanif et al.^67^, and Khan et al.^59^. PCGDP^2^ and HCI show a significant negative impact on *CO*_2_ emissions, with coefficients of 0.065 and 0.227 with random effects, and 0.075 and 0.162 with fixed effects, respectively. The diagnostic checks of the estimated random effects and fixed effects models are reported at the rare part of Table 7. The results indicate that the adjusted r-squared values are 0.842 and 0.911 for random effects and fixed effects models, respectively. The corresponding p-values of the F-statistics are significant at the 1% level, implying the joint significance of the augmented variables. Finally, the results of the Jarque–Bera test indicate that the residuals are normally distributed across both models.

## Discussion

Following the controversial issue of the environment that has seriously threatened the goal of sustainable development, especially in developing economies, in the present study, we raised three contemporary research questions in the context of South Asia as follows: First, is the EKC hypothesis valid in the South Asian context? Second, what is the scale and magnitude of the effects of overpopulation on existing *CO*_2_ emissions? Third, is there any long-run memory between energy consumption, overpopulation, *CO*_2_ emissions, and growth indicators in South Asia? To find rational answers to the research questions and specify precise policy implications, we designed our study using the celebrated Environmental Kuznets Curve (EKC) framework. The study employed a set of pollutant and macroeconomic indicators and datasets ranging from 1972 to 2021 for five South Asian countries (Bangladesh, India, Nepal, Pakistan, and Sri Lanka). The initial descriptive statistics (Table 2, Figs. 1 and 2) indicate that *CO*_2_ emissions and per capita GDP significantly increased over the period under review; however, the level of energy consumption, population density, and human capital index also rose proportionately over time. Further analysis revealed that *CO*_2_ emissions have a long-run relationship with energy consumption, per capita GDP, population density, and human capital index (Table 5). These results imply that the proportion of economic growth has been substantively associated with an increase in environmental degradation caused by *CO*_2_ emissions, energy consumption, and population density in South Asia. Moreover, our findings reveal that there is unidirectional causality running from energy consumption, per capita GDP, and the square of per capita GDP (Table 6), laying the empirical groundwork for the assessment of the EKC hypothesis.

Our final results obtained from the estimation of random effects and fixed effects models (Table 7) confirm the validity of an inverted U-shaped curve between economic expansion and environmental degradation. The existence of the EKC implies that South Asian countries can actually grow out of pollution in the coming years. These findings are in line with the observations of some recent studies. For example, Jóźwik et al.^4^ found a long run equilibrium relationship between environmental degradation, energy consumption, and economic growth in South Asian countries. Similar findings are reported by Vural^97^, who found the validity of the EKC in the case of 6 Association of Southeast Asian nations (ASEAN) and 8 South African nations, respectively. The result that energy consumption is a key source of $CO_{2}$ emissions is in line with previous empirical literature conducted on the nexus between $CO_{2}$ emissions and energy consumption. For example, Heidari et al.^98^ revealed that energy consumption leads to an increase in $CO_{2}$ emissions in 5 ASEAN countries. However, Jiang et al.^99^ found that effective energy system analysis has an emission-lessening impact in China. We find that population density increases $CO_{2}$ emissions. The literature on the role of population growth, especially urbanization, in the context of $CO_{2}$ emissions, is inconclusive. For instance, Anwar et al.^100^ reported the positive impact of population on $CO_{2}$ emissions. Similar findings are reached by Raihan et al.^101^. Moreover, Lee et al.^102^ indicated that $CO_{2}$ emissions rise because of rapid urbanization. On the other hand, there are studies, who state that an increase in population density reduces $CO_{2}$ emissions. Ali et al.^103^ found that $CO_{2}$ emissions level decreased in Singapore because of urbanization. Wang et al.^104^ state the negative association between urbanization and $CO_{2}$ emissions in OECD countries. Likewise, Li et al.^105^ show that $CO_{2}$ emissions reduce in anticipation to urbanization. Our findings that human capital is negatively associated with $CO_{2}$ emissions agree with previous literature. For instance, Mahmood et al.^106^ reached the conclusion that improvement in human capital results in mitigation of $CO_{2}$ emissions. Sapkota and Bastola^107^ are of the view that expansion in human capital reduces pollution level through the adoption of cleaner production machinery.

We discuss the potential implications of the above findings in the context of economic, social, political, and technological perspectives. Although energy is an unmet need of an economy as it enters as a crucial input in production functions, it damages the environmental quality by increasing the level of $CO_{2}$ emissions^108,109^. In South Asia, the main sources of energy are non-renewable (e.g., oil and gas), and governments struggle to tap renewable energy sources despite their immense potential in all the countries to varying degrees. Politically, policymakers in South Asian countries need to devise policies that are environmentally friendly and encourage renewable energy use in all sectors by providing incentives and investing in the generation of renewable energy. As the government has scarce resources, a public–private partnership could be one option to address the environmental challenge. The financial sector needs to be encouraged to a divert large number of resources into granting loans to support environmentally friendly projects. The governments may embrace international renewable energy cooperation to gradually adopt renewable energy technology. On the other hand, regulations, including the imposition of carbon taxes and tariffs, can be tightened to combat environmental issues. On the economic front, these nations need to stimulate real income by pursuing prudent fiscal and monetary policies with a focus on investment in human capital, which is key to accelerating real income. From a social perspective, policymakers need to address social behavior or activities that are not environmentally friendly. This can be achieved through massive public awareness campaigns that focus on cultural beliefs and social lifestyles that lead to environmental problems. Technologically, governments should invest in new discoveries and embrace foreign investment in the areas of renewable energy resources. Currently, one of the biggest obstacles in the way of adopting renewable energy is its higher cost relative to traditional sources of energy. Mobilizing resources towards research and development could substantially decrease the per-unit cost of production of renewable energy relative to non-renewable energy and hence address environmental issues^110^.

## Conclusion and policy implications

Contributing to the realization of SDG-12 and SDG-13 that call for prompt actions to sustain environmental quality and reduce the impact of climate change on humans and their surroundings, the present study aims to investigate the effects of per capita GDP, overpopulation, human capital index, and energy consumption on CO_2_ emissions. In a bid to add to the contemporary body of knowledge, we designed our inquiry to focus on one of the most vulnerable research contexts—South Asia, Bangladesh, India, Nepal, Pakistan, and Sri Lanka, in particular. The primary objective of the study is to explore how overpopulation and human interaction contribute to the accelerating environmental degradation in South Asia. To that end, we use a set of panel data over the period from 1972 to 2021 and frame our investigation on the EKC assumption. To test the EKC hypothesis, we use a set of panel econometric techniques that are capable of solving potential problems of time instability and heterogeneity.

Initial statistical results reveal that there is a significant cross-sectional dependence among the cited South Asian countries, implying that they follow similar patterns of environmental behavior, growth strategy, and heating power consumption habits. Further, the results of the panel unit root analysis demonstrate that the variables are level-stationary and thus follow the I(0) series. Additionally, the dynamic panel cointegration results obtained from Westerlund’s approach confirm that *CO*_2_ emissions, per capita GDP, the square of per capita GDP, population density, energy consumption, and human capital index have a long-run relationship. It implies that the explanatory variables differently affect *CO*_2_ emissions in the long run, suggesting that we delve into their causal nexus. To that faith, the study employs the dynamic panel causality test of Dumitrescu and Hurlin and observes that energy consumption, per capita GDP, and the square of per capita GDP significantly cause CO_2_ emissions in South Asia, while the results fail to document any feedback response. Interestingly, the results indicate that concurrent CO2 emissions strongly cause a higher population density and human capital index in the region. Furthermore, the baseline results obtained from the random effects model—that is, preferred over the fixed effects model by the Hausman test—indicate that per capita GDP has an incremental effect on *CO*_2_ emissions; however, the square of per capita GDP posits reduction effects on the subject. Our results lend statistical support for the validity of the EKC hypothesis in South Asia. Additionally, the results indicate that energy consumption and population density are positively associated with *CO*_2_ emissions, while the human capital index reduces *CO*_2_ emissions in South Asia.

### Policy implications

On careful scrutiny of the empirical conclusions drawn, several and yet specific policy implications can be highlighted as follows:

i. *Technological innovation* The findings suggest that the existing growth-targeting regime in South Asia is highly associated with higher CO_2_ emissions. Governments need to ensure that sufficient resources are allocated to invest in technological innovations that generate lower emissions in the production of goods. Promotion and institutionalization of hybrid vehicles instead of existing gasoline and diesel-consuming vehicles, both on private and public transportation, would be in high favor of improving environmental quality. This can be achieved without advocating for a growth suppression strategy.
ii. *Regulatory intervention* The existing consumption pattern in South Asia is regulatory-free. The consumption of heating materials that significantly contribute to environmental degradation is uncontrolled. Public baths, households, and firms consume non-standard heating materials. Therefore, consolidated regulatory frameworks that promote and impose green consumption behavior is necessary.
iii. *Human capital development* The findings suggest that governments need to enhance the existing capacity and knowledge of human capital in South Asia with respect to green environmental behavior. Allocating more resources towards education brings improvements in environmental quality, as aspired to by the Paris Agreement. Massive awareness campaigns among the community, including students, to use green energy instead of traditional methods of energy are suggested, as people’s acceptance or rejection of a policy determines the success or failure of that policy.
iv. *Renewable energy* The minimum reliance on fossil fuel energy sources should be prioritized to minimize the negative impact of energy consumption on *CO*_2_ emissions. Funding of renewable and clean energy sources can help in improving energy security, transitioning towards a low-carbon economy, and achieving sustainability. It is believed that developing renewable energy resources such as solar, wind, and hydroelectric power plants will replace non-renewable sources of energy. The larger economies of the region, Pakistan and India, should take the lead in making the region environmentally friendly by implementing strict environmental regulations without lowering income and output levels.

### Limitations of the study

This study suffers from two key limitations: First, due to the unavailability of the required data, we only focused on five South Asian countries. Upon the availability of data, future studies may follow a similar framework by including the remaining three countries, such as Afghanistan, Bhutan, and the Maldives, in their analysis. Second, due to multicollinearity issues, the study did not account for the spillovers of exogenous variables that may influence contemporary environmental degradation. Future studies may overcome this challenge by incorporating greenwashing, financial technologies, and institutional quality indicators to examine the validity of the EKC assumption in the recipient panel.

## Data Availability

Data is available from correspondence author on reasonalbe request.

## Abbreviations

CO2: Carbon dioxide emissions
CD: Cross-sectional dependence
CIPS: Cross-sectional Im, Pesaran, and Shin
EG: Economic growth
EF: Ecological footprint
EKC: Environmental Kuznets curve
ENGU: Energy use
EX: Export
FI: Financial inclusion
GHGs: Greenhouse gases
GDPPC: GDP per capita
HCI: Human capital index
HDI: Human development index
IT: Intra-regional trade
LM: Lagrange multiplier
NRE: Non-renewable energy
POPD: Population density
RET: Renewable energy transition
SA: South Asia
TR: Trade openness

## References

[1] Le HP, Ozturk I (2020). The impacts of globalization, financial development, government expenditures, and institutional quality on CO_2_ emissions in the presence of environmental Kuznets curve. Environ. Sci. Pollut. Res..

[2] Apergis N, Payne JE (2014). Renewable energy, output, CO_2_ emissions, and fossil fuel prices in Central America: Evidence from a nonlinear panel smooth transition vector error correction model. Energy Econ..

[3] Husnain MIU, Haider A, Khan MA (2021). Does the environmental Kuznets curve reliably explain a developmental issue?. Environ. Sci. Pollut. Res..

[4] Jóźwik B, Kyophilavong P, Dash AK, Gavryshkiv AV (2022). Revisiting the environmental Kuznets curve hypothesis in South Asian Countries: The role of energy consumption and trade openness. Energies.

[5] Thapa, G. “Rural poverty reduction strategy for South Asia,” in *Economic Growth, Economic Performance and Welfare in South Asia* (2005).

[6] Rasul G (2021). Twin challenges of COVID-19 pandemic and climate change for agriculture and food security in South Asia. Environ. Chall..

[7] Asadullah MN, Savoia A, Sen K (2020). Will South Asia achieve the sustainable development goals by 2030? Learning from the MDGs experience. Soc. Indic. Res..

[8] Rashid Gill A, Viswanathan KK, Hassan S (2018). The environmental Kuznets curve (EKC) and the environmental problem of the day. Renew. Sustain. Energy Rev..

[9] Ahmad M, Zhao ZY (2018). Empirics on linkages among industrialization, urbanization, energy consumption, CO_2_ emissions and economic growth: A heterogeneous panel study of China. Environ. Sci. Pollut. Res..

[10] Rahman MM (2020). Environmental degradation: The role of electricity consumption, economic growth and globalisation. J. Environ. Manage..

[11] Apergis N, Payne JE (2009). CO_2_ emissions, energy usage, and output in Central America. Energy Policy.

[12] Dogadn E, Aslan A (2017). Exploring the relationship among CO_2_ emissions, real GDP, energy consumption and tourism in the EU and candidate countries: Evidence from panel models robust to heterogeneity and cross-sectional dependence. Renew. Sustain. Energy Rev..

[13] Apergis N, Christou C, Gupta R (2017). Are there environmental Kuznets curves for US state-level CO_2_ emissions?. Renew. Sustain. Energy Rev..

[14] Rahman MM, Saidi K, BenM barek M (2020). Economic growth in South Asia: The role of CO_2_ emissions, population density and trade openness. Heliyon.

[15] Rahman MM, Nepal R, Alam K (2021). Impacts of human capital, exports, economic growth and energy consumption on CO_2_ emissions of a cross-sectionally dependent panel: Evidence from the newly industrialized countries (NICs). Environ. Sci. Policy.

[16] Haider A, Rankaduwa W, ul Husnain MI, Shaheen F (2022). Nexus between agricultural land use, economic growth and N_2_O emissions in Canada: Is there an environmental Kuznets curve?. Sustainability.

[17] ul Husnain MI, Beyene SD, Aruga K (2023). Investigating the energy-environmental Kuznets curve under panel quantile regression: a global perspective. Environ. Sci. Pollut. Res..

[18] Nasrullah N, ul Husnain MI, Khan MA (2023). The dynamic impact of renewable energy consumption, trade, and financial development on carbon emissions in low-, middle-, and high-income countries. Environ. Sci. Pollut. Res..

[19] Murshed M, Dao NTT (2022). Revisiting the CO_2_ emission-induced EKC hypothesis in South Asia: The role of Export Quality Improvement. GeoJournal.

[20] Mehmood U, Tariq S, ul Haq Z, Azhar A, Mariam A (2022). The role of tourism and renewable energy towards EKC in South Asian countries: fresh insights from the ARDL approach. Cogent. Soc. Sci..

[21] Ali M, Tariq M, Azam Khan M (2022). Re-examining the Kuznets curve hypothesis for South Asian Countries: New evidences. J. Asian Afr. Stud..

[22] Tan YL, Yiew TH, Lau LS, Tan AL (2022). Environmental Kuznets curve for biodiversity loss: Evidence from South and Southeast Asian countries. Environ. Sci. Pollut. Res..

[23] Sadiq M, Kannaiah D, Yahya Khan G, Shabbir MS, Bilal K, Zamir A (2023). Does sustainable environmental agenda matter? The role of globalization toward energy consumption, economic growth, and carbon dioxide emissions in South Asian countries. Environ. Dev. Sustain..

[24] DeJong DN, Nankervis JC, Savin NE, Whiteman CH (1992). The power problems of unit root test in time series with autoregressive errors. J. Econom..

[25] Narayan PK, Smyth R (2009). Multivariate granger causality between electricity consumption, exports and GDP: Evidence from a panel of Middle Eastern countries. Energy Policy.

[26] Aslanidi, N. Environmental Kuznets curves for carbon emissions: A critical survey. in *Handbook of Environmental Policy* (2011), pp. 205–224.

[27] Hsiao C. *Analysis of Panel Data* (2022).

[28] Pesaran MH, Smith R (1995). Estimating long-run relationships from dynamic heterogeneous panels. J. Econom..

[29] Brännäs K, Granger CWJ, Teräsvirta T, Brannas K, Terasvirta T (1994). Modelling non-linear economic relationships. Scand. J. Econ..

[30] Dumitrescu EI, Hurlin C (2012). Testing for granger non-causality in heterogeneous panels. Econ. Model..

[31] Dogan E, Seker F (2016). Determinants of CO_2_ emissions in the European Union: The role of renewable and non-renewable energy. Renew. Energy.

[32] Driscoll JC, Kraay AC (1998). Consistent covariance matrix estimation with spatially dependent panel data. Rev. Econ. Stat..

[33] van den Bergh JCJM, Botzen WJW (2018). Global impact of a climate treaty if the Human Development Index replaces GDP as a welfare proxy. Clim. Policy.

[34] Costa L, Rybski D, Kropp JP (2011). A human development framework for CO_2_ reductions. PLoS One.

[35] Gorus MS, Aydin M (2019). The relationship between energy consumption, economic growth, and CO2 emission in MENA countries: Causality analysis in the frequency domain. Energy.

[36] Grossman, G. & Krueger, A. *Environmental Impacts of a North American Free Trade Agreement* (1991). 10.3386/w3914.

[37] Panayotou T (1994). Empirical tests and policy analysis of environmental degradation at different stages of economic development. Pacific Asian J. Energy.

[38] Naradda Gamage SK, Hewa Kuruppuge R, ul Haq I (2017). “Energy consumption, tourism development, and environmental degradation in Sri Lanka”, Energy Sources. Part B Econ. Plan. Policy.

[39] Bastola U, Sapkota P (2015). Relationships among energy consumption, pollution emission, and economic growth in Nepal. Energy.

[40] Mukhtarov S, Humbatova S, Seyfullayev I, Kalbiyev Y (2020). The effect of financial development on energy consumption in the case of Kazakhstan. J. Appl. Econ..

[41] Ding Q, Khattak SI, Ahmad M (2021). Towards sustainable production and consumption: Assessing the impact of energy productivity and eco-innovation on consumption-based carbon dioxide emissions (CCO_2_) in G-7 nations. Sustain. Prod. Consum..

[42] Ali S, Dogan E, Chen F, Khan Z (2021). International trade and environmental performance in top ten-emitters countries: The role of eco-innovation and renewable energy consumption. Sustain. Dev..

[43] Sarkodie SA, Strezov V (2019). Effect of foreign direct investments, economic development and energy consumption on greenhouse gas emissions in developing countries. Sci. Total Environ..

[44] Nathaniel SP, Adeleye N (2021). Environmental preservation amidst carbon emissions, energy consumption, and urbanization in selected african countries: Implication for sustainability. J. Clean. Prod..

[45] Wang S, Wang J, Li S, Fang C, Feng K (2019). Socioeconomic driving forces and scenario simulation of CO_2_ emissions for a fast-developing region in China. J. Clean. Prod..

[46] Shahzad U (2020). Environmental taxes, energy consumption, and environmental quality: Theoretical survey with policy implications. Environ. Sci. Pollut. Res..

[47] Omer, A.M. Energy use and environmental impacts: A general review, in *Advances in Energy Research* (2014), 1–38.

[48] Acheampong AO (2018). Economic growth, CO_2_ emissions and energy consumption: What causes what and where?. Energy Econ..

[49] Dietz T, Rosa EA (1994). Rethinking the environmental impacts of population, affluence and technology. Hum. Ecol. Rev..

[50] Brian, W. L. O’Neill, C. & Landis MacKellar, F. *Population and Climate Change* (2001).

[51] Begum RA, Sohag K, Abdullah SMS, Jaafar M (2015). CO_2_ emissions, energy consumption, economic and population growth in Malaysia. Renew. Sustain. Energy Rev..

[52] Rehman A (2023). Globalization and renewable energy use: How are they contributing to upsurge the CO_2_ emissions? A global perspective. Environ. Sci. Pollut. Res..

[53] Jahanger A, Hossain MR, Onwe JC, Ogwu SO, Awan A, Balsalobre-Lorente D (2023). Analyzing the N-shaped EKC among top nuclear energy generating nations: A novel dynamic common correlated effects approach. Gondwana Res..

[54] Ganda F (2022). The environmental impacts of human capital in the BRICS economies. J. Knowl. Econ..

[55] Çamkaya S, Karaaslan A, Uçan F (2023). Investigation of the effect of human capital on environmental pollution: empirical evidence from Turkey. Environ. Sci. Pollut. Res..

[56] Abdouli M, Omri A (2021). Exploring the nexus among FDI inflows, environmental quality, human capital, and economic growth in the mediterranean region. J. Knowl. Econ..

[57] Murshed M, Nurmakhanova M, Al-Tal R, Mahmood H, Elheddad M, Ahmed R (2022). Can intra-regional trade, renewable energy use, foreign direct investments, and economic growth mitigate ecological footprints in South Asia?. Energy Sources Part B Econ. Plan. Policy.

[58] Majumder SC, Voumik LC, Rahman MH, Rahman MM, Hossain MN (2023). A quantile regression analysis of the impact of electricity production sources on CO_2_ emission in South Asian Countries. Strateg. Plan. Energy Environ..

[59] Khan MB, Saleem H, Shabbir MS, Huobao X (2022). The effects of globalization, energy consumption and economic growth on carbon dioxide emissions in South Asian countries. Energy Environ..

[60] Lau HC, Zhang K, Bokka HK, Ramakrishna S (2022). A review of the status of fossil and renewable energies in Southeast Asia and its implications on the decarbonization of ASEAN. Energies.

[61] Amin N, Song H, Khan ZA (2022). Dynamic linkages of financial inclusion, modernization, and environmental sustainability in South Asia: a panel data analysis. Environ. Sci. Pollut. Res..

[62] Raza SMF, Ali I, Malik MY, Ahmad M, Abidin SZU, Masood S (2022). Renewable energy consumption towards environmental sustainability: evidence of EKC hypothesis in the framework of economic growth and CO_2_ emissions. PalArch’s J. Archaeol. Egypt/egyptol..

[63] Sharma R, Shahbaz M, Sinha A, Vo XV (2021). Examining the temporal impact of stock market development on carbon intensity: Evidence from South Asian countries. J. Environ. Manage..

[64] Jingpeng L, Ullah A, Raza SA, Ahmed M (2023). Nonlinear and nonparametric causal relationship between financial inclusion and sustainable environment in South Asia. Environ. Sci. Pollut. Res..

[65] Fong LS, Salvo A, Taylor D (2020). Evidence of the environmental Kuznets curve for atmospheric pollutant emissions in Southeast Asia and implications for sustainable development: A spatial econometric approach. Sustain. Dev..

[66] Sattar A, Tolassa TH, Hussain MN, Ilyas M (2022). Environmental effects of China’s overseas direct investment in South Asia. SAGE Open.

[67] Hanif S, Nawaz A, Hussain A, Bhatti MA (2022). Linking non renewable energy, renewable energy, globalization and CO_2_ emission under EKC hypothesis: Evidence from ASEAN-6 countries through advance panel estimation. Pakistan J. Humanit. Soc. Sci..

[68] Rahman MM, Alam K (2022). CO_2_ emissions in Asia-pacific region: Do energy use, economic growth, financial development, and international trade have detrimental effects?. Sustainability.

[69] Wangzhou K, Wen JJ, Wang Z, Wang H, Hao C, Andlib Z (2022). Revealing the nexus between tourism development and CO_2_ emissions in Asia: Does asymmetry matter?. Environ. Sci. Pollut. Res..

[70] Sampene AK, Li C, Khan A, Agyeman FO, Brenya R, Wiredu J (2023). The dynamic nexus between biocapacity, renewable energy, green finance, and ecological footprint: evidence from South Asian economies. Int. J. Environ. Sci. Technol..

[71] Mikayilov JI, Galeotti M, Hasanov FJ (2018). The impact of economic growth on CO_2_ emissions in Azerbaijan. J. Clean. Prod..

[72] Jahanger A, Zaman U, Hossain MR, Awan A (2023). Articulating CO_2_ emissions limiting roles of nuclear energy and ICT under the EKC hypothesis: An application of non-parametric MMQR approach. Geosci. Front..

[73] Alaganthiran JR, Anaba MI (2022). The effects of economic growth on carbon dioxide emissions in selected Sub-Saharan African (SSA) countries. Heliyon.

[74] Jahanger A, Usman M, Balsalobre-Lorente D (2022). Linking institutional quality to environmental sustainability. Sustain. Dev..

[75] Aslam B (2021). The nexus of industrialization, GDP per capita and CO2 emission in China. Environ. Technol. Innov..

[76] Adeleye BN, Osabohien R, Lawal AI, de Alwis T (2021). Energy use and the role of per capita income on carbon emissions in African countries. PLoS One.

[77] Jahanger A, Zubair Chishti M, Chukwuma Onwe J, Awan A (2022). How far renewable energy and globalization are useful to mitigate the environment in Mexico? Application of QARDL and spectral causality analysis. Renew. Energy.

[78] Ivanovski K, Awaworyi Churchill S, Nuhu AS (2020). Modelling the Australian J-curve: An ARDL cointegration approach. Econ. Pap..

[79] Jin T, Kim J (2018). What is better for mitigating carbon emissions; Renewable energy or nuclear energy? A panel data analysis. Renew. Sustain. Energy Rev..

[80] Sudhakara Reddy B, Assenza GB (2009). The great climate debate. Energy Policy.

[81] Zhu M (2022). The role of human capital and environmental protection on the sustainable development goals: New evidences from Chinese economy. Econ. Res. Istraz..

[82] World Bank, “World development indicators | data,” *World Development Indicators*, 2023. Retrieved 23 Mar 2020 from https://databank.worldbank.org/source/world-development-indicators.

[83] Rahman MM (2017). Do population density, economic growth, energy use and exports adversely affect environmental quality in Asian populous countries?. Renew. Sustain. Energy Rev..

[84] Zoundi Z (2017). CO_2_ emissions, renewable energy and the environmental Kuznets curve, a panel cointegration approach. Renew. Sustain. Energy Rev..

[85] Rahman MM, Vu XB (2020). The nexus between renewable energy, economic growth, trade, urbanisation and environmental quality: A comparative study for Australia and Canada. Renew. Energy.

[86] Breusch TS, Pagan AR (1980). The lagrange multiplier test and its applications to model specification in econometrics. Rev. Econ. Stud..

[87] Pesaran MH (2021). General diagnostic tests for cross-sectional dependence in panels. Empir. Econ..

[88] Maddala GS, Wu S (1999). A comparative study of unit root tests with panel data and a new simple test. Oxf. Bull. Econ. Stat..

[89] Pesaran MH (2007). A simple panel unit root test in the presence of cross-section dependence. J. Appl. Econom..

[90] Kao C (1999). Spurious regression and residual-based tests for cointegration in panel data. J. Econom..

[91] Pedroni P (2004). Panel cointegration: Asymptotic and finite sample properties of pooled time series tests with an application to the PPP hypothesis. Econom. Theory.

[92] Pedroni P (1999). Critical values for cointegration tests in heterogeneous panels with multiple regressors. Oxf. Bull. Econ. Stat..

[93] Westerlund J (2005). New simple tests for panel cointegration. Econom. Rev..

[94] Barassi MR (2006). Microeconometrics: Methods and applications. Econ. J..

[95] Hausman JA (1978). Specification tests in econometrics. Econometrica.

[96] Sinaga O, Saudi MHM, Roespinoedji D, Jabarullah NH (2019). Environmental impact of biomass energy consumption on sustainable development: Evidence from ARDL bound testing approach. Ekoloji.

[97] Vural G (2020). How do output, trade, renewable energy and non-renewable energy impact carbon emissions in selected Sub-Saharan African Countries?. Resour. Policy.

[98] Heidari H, Turan Katircioǧlu S, Saeidpour L (2015). Economic growth, CO_2_ emissions, and energy consumption in the five ASEAN countries. Int. J. Electr. Power Energy Syst..

[99] Jiang T, Yu Y, Jahanger A, Balsalobre-Lorente D (2022). Structural emissions reduction of China’s power and heating industry under the goal of ‘double carbon’: A perspective from input-output analysis. Sustain. Prod. Consum..

[100] Anwar A, Younis M, Ullah I (2020). Impact of urbanization and economic growth on CO_2_ emission: A case of far east Asian countries. Int. J. Environ. Res. Public Health.

[101] Raihan A, Chandra Voumik L (2022). Carbon emission dynamics in India due to financial development, renewable energy utilization, technological innovation, economic growth, and urbanization. J. Environ. Sci. Econ..

[102] Lee CC, Zhou B, Yang TY, Yu CH, Zhao J (2023). The impact of urbanization on CO_2_ emissions in China: The key role of foreign direct investment. Emerg. Mark. Financ. Trade.

[103] Ali HS, Abdul-Rahim A, Ribadu MB (2017). Urbanization and carbon dioxide emissions in Singapore: Evidence from the ARDL approach. Environ. Sci. Pollut. Res..

[104] Wang WZ, Liu LC, Liao H, Wei YM (2021). Impacts of urbanization on carbon emissions: An empirical analysis from OECD countries. Energy Policy.

[105] Li J, Huang X, Kwan MP, Yang H, Chuai X (2018). The effect of urbanization on carbon dioxide emissions efficiency in the Yangtze River Delta, China. J. Clean. Prod..

[106] Mahmood N, Wang Z, Hassan ST (2019). Renewable energy, economic growth, human capital, and CO_2_ emission: An empirical analysis. Environ. Sci. Pollut. Res..

[107] Sapkota P, Bastola U (2017). Foreign direct investment, income, and environmental pollution in developing countries: Panel data analysis of Latin America. Energy Econ..

[108] Apergis N, Payne JE (2010). Renewable energy consumption and economic growth: Evidence from a panel of OECD countries. Energy Policy.

[109] Paramati SR, Apergis N, Ummalla M (2018). Dynamics of renewable energy consumption and economic activities across the agriculture, industry, and service sectors: Evidence in the perspective of sustainable development. Environ. Sci. Pollut. Res..

[110] Ehigiamusoe KU, Dogan E (2022). The role of interaction effect between renewable energy consumption and real income in carbon emissions: Evidence from low-income countries. Renew. Sustain. Energy Rev..

